# Primary Carcinoma of the Gallbladder

**DOI:** 10.1155/1991/92731

**Published:** 1991

**Authors:** John A. Paraskevopoulos, Ashley R. Dennison, Alan G. Johnson

**Affiliations:** University Department of Surgery Floor K Royal Hallamshire Hospital Glossop Road Sheffield S10 2JF UK

## Abstract

Carcinoma of the gallbladder is a relatively rare malignancy which is difficult to diagnose. The advent of improved imaging methods and the expansion of interventional radiology however, combined with advances in surgical technique, has produced a change in attitude towards this tumour. The available world literature since 1960 has been reviewed and is presented in this article. However, whilst the outlook for diagnosis and treatment is improving, clearly the association with cholelithiasis (between 45% and 100%), is a cause for concern particularly with the advent of treatments (lithotripsy, percutaneous gallstone extraction) which leave gall bladder mucosa and residual fragments of stone *in situ* .


*HPB Surgery*, 1991, Vol. 4, pp 277–289
Reprints available directly from the publisher
Photocopying permitted by license only

© 1991 Harwood Academic Publishers GmbH
Printed in the United Kingdom

### PRIMARY CARCINOMA OF THE GALLBLADDER

JOHN A. PARASKEVOPOULOS, ASHLEY R. DENNISON and ALAN G. JOHNSON

*University Department of Surgery, Floor K, Royal Hallamshire Hospital, Glossop Road, Sheffield S10 2JF, UK*

*(Received 27 March 1991)*

Carcinoma of the gallbladder is a relatively rare malignancy which is difficult to diagnose. The advent of improved imaging methods and the expansion of interventional radiology however, combined with advances in surgical technique, has produced a change in attitude towards this tumour. The available world literature since 1960 has been reviewed and is presented in this article. However, whilst the outlook for diagnosis and treatment is improving, clearly the association with cholelithiasis (between 45% and 100%), is a cause for concern particularly with the advent of treatments (lithotripsy, percutaneous gallstone extraction) which leave gall bladder mucosa and residual fragments of stone *in situ*.

KEY WORDS: Gallbladder carcinoma, prognosis, treatment

### INTRODUCTION

Primary carcinoma of the gallbladder was first described by Maximilian Stoll in 1777 and is an uncommon neoplasm encountered in about 1% of cholecystectomy operations and about 1% of autopsies in the United Kingdom[1,2], but it is the most common of the biliary tract malignancies resulting in approximately 6,500 deaths per year in the USA. It predominantly affects women with a peak incidence in the 70 to 80 year old age group with female to male ratio of approximately 3–4:1, although ratios as high as 8:1 have been reported[3,4].

In the early stages of the disease symptoms are extremely uncommon and if they do occur, are usually due to the co-existence of cholecystitis, cholelithiasis or complications of associated benign biliary disease. Severe local or generalised symptoms occur only when the disease is advanced and has spread beyond the gallbladder. Sixteen percent of gallbladder carcinomas are found incidentally during examination of the routine cholecystectomy specimens[5].

Ninety eight percent of malignant tumours of the gallbladder are carcinomas and they are generally classified (according to their histologic differentiation) into: adenocarcinoma, squamous cell carcinoma, adenosquamous carcinoma and oat cell carcinoma[5]. The means of spread of these carcinomas may be lymphatic, vascular, intraperitoneal, neural, intraductal or by direct extension[6]. Prognosis is related to the extent of the disease and consequently in 1976 a staging and grading system was introduced by Nevin *et al*[7]. They suggested five stages: Stage I—intramucosal;

Address correspondence to: John A. Paraskevopoulos, University Department of Surgery, Floor K, Royal Hallamshire Hospital, Glossop Road, Sheffield S10 2JF, UK

Stage II—involvement of mucosa and muscularis; Stage III—involvement of all three layers; Stage IV—involvement of all three layers and the cystic lymph node; Stage V—involvement of liver. The histological grades were: Grade I (well differentiated), Grade II (moderately differentiated) and Grade III (poorly differentiated).

Despite recent advances in surgery of the liver and the biliary tract, there has been no dramatic improvement in the prognosis of the primary carcinoma of the gallbladder. Five year survival rates of 1.4 to 4.1% are generally reported[8], although in a recent review a five year survival of 12% was found[9]. This poor outlook is mainly due to the lack of an effective means of pre-operative diagnosis (despite the use of ultrasound, computerised tomography and magnetic resonance imaging) although interventional radiological techniques such as arteriography seem very promising in assessing the lesion after the diagnosis is suspected.

We have reviewed the available literature on this subject over the last 30 years and in this article have described the main findings of the 42 major series[4,6–47].

### AETIOLOGICAL FACTORS

While the aetiology of carcinoma of the gallbladder remains obscure, it is likely that more than one factor is important in the pathogenesis. The co-existence of these conditions with gallbladder carcinoma and the risk of carcinoma transformation are shown in Table 1. Review of the literature reveals that although cholelithiasis, genetic factors, malignant transformation of benign neoplasms of the gallbladder, congenital abnormalities, bacterial infections, occupation, xanthogranulomatous cholecystitis, chronic inflammatory bowel disease and previous biliary surgery have been all noted in association with carcinoma of the gallbladder, gallstones remain the sole major known risk factor.

*Cholelithiasis* It is estimated that 10 to 20 percent of the adult population in developed countries have gallstones with a higher prevalence in women, and obese and elderly patients[48]. Although gallstones are found in 45 to 100% of patients with gallbladder carcinoma (Table 2), autopsy studies have shown that the overall incidence of carcinoma of the gallbladder in all patients with cholelithiasis (irrespective of age) is only 1 to 3 percent[49]. In an epidemiological study from Minnesota where 2583 known patients with gallstones were followed for the development of gastrointestinal malignancies, it was found that the risk for gallbladder carcinoma was increased threefold, but the increase was only significant in men[50]. According to Diehl, the risk of cancer developing among patients with untreated cholelithiasis was estimated to be between 0.2 and 0.5 percent over a 20 year period[51]. Diehl has also shown a strong relationship between gallstone size and gallbladder carcinoma, larger gallstones being associated with an increased risk of carcinoma; for stones of 2.0 to 2.9 cm in diameter, the odds ratio (versus stone size of < 1 cm) was 2.4, whereas for stones of 3 cm or larger, the ratio was 10.1[52]. Similar figures have been presented by Lowenfels *et al.* according to whom, the relative risk for gallbladder carcinoma in people with stones of > 3cm in diameter is 9.2 compared to stones of < 1 cm[53]. In this study it was estimated that 30% of all gallbladder carcinomas are associated with large ( > 3 cm) stones and that the annual rate of gallstone growth is 2 mm[53]. This is particularly relevant to selection criteria for extracorporeal lithotripsy which at present is used for small stones. Gallbladder carcinoma is

**Table 1** Aeitiological factors

| *Factors* | *Associated finding (%)* | *Relative risk of gall-bladder carcinoma* | *References* |
|---|---|---|---|
| 1. *Gallstones* | >80 | ↑ 3 only in men; 0.2–0.5% (over 20 years in both sexes) | (5,50,51) |
| *Size of gallstones* | — | ≥3 cm: ≤1 cm = 10.1 | (52,53) |
| *Type of gallstones* | | | (5) |
| 2. *Genetic* | | | (50) |
| - American Indians | — | — | |
| - Black American women | | | |
| - Swedish men and women | | | |
| 3. *Congenital cysts* | — | ↑ 20 fold | (54,55) |
| 4. *Familial anomalous pancreatobiliary duct union* | — | — | (56) |
| 5. *Adenoma* | 19 | — | (57,58) |
| 6. *Adenomyomatosis* | — | — | (61) |
| 7. *Diffuse calcification* (= porcelain gallbladder) | 10–25 | — | (62) |
| 8. *Bacterial infections* | — | ↑ 6 fold | (63) |
| 9. *Xanthogranulomatous cholecystitis* | 8.6 | — | (65) |
| 10. *Occupation* (rubber industry) | — | — | (66) |
| 11. *Chronic inflammatory bowel disease* | 13 | — | (67) |
| 12. *Previous biliary surgery* | — | — | (49) |

usually associated with cholesterol type stones which are formed by precipitation (not secondary to pre-existing stones or infection)[5,46]. Whilst the aetiological relationship of gallstones to carcinoma of the gallbladder remains incompletely understood, chronic irritation is believed to be the major promoting factor for neoplastic transformation[5].

*Genetic factors* There is an increased incidence of carcinoma of the gallbladder in certain racial and ethnic groups. In a case control study gallstones were associated with an increased risk for gallbladder carcinoma in American Indians (men and women), black American women and Swedish men and women[50]. Unusually high rates of gallbladder carcinoma are also found among Hispanic Americans, possibly due to racial intermixture[5].

*Congenital anomalies* Cystic dilatation of the biliary tree predisposes to extrahepatic bile duct carcinoma with a 2.5 percent incidence[54]. The incidence of carcinoma in choledochal cysts is 20 times greater than the incidence of bile duct carcinoma in the general population with adenocarcinoma being the most commonly found histological variety[55]. Anomalous pancreatic biliary duct union without the presence of choledochal cyst has been recently reported to show an increased association with gallbladder carcinoma[56]. In these cases it is suggested

**Table 2** Carcinoma of the gallbladder review of the world literature: 1960–1990

| *References* | *No of cases* | *Mean Age (years)* | *M:F* | *Cholelithiasis (%)* | *5 year survival (%)* |
|---|---|---|---|---|---|
| (10) | 47 | 63 | 1:3 | — | 4.25 |
| (11) | 70 | 67.9 | 1:3.4 | 54.3 | 1.43 |
| (12) | 132 | 61 | 1:3.5 | 89 | 2 |
| (6) | 151 | — | — | — | — |
| (13) | 76 | — | 1:2 | 90 | 4 |
| (14) | 52 | 65 | 1:2.5 | 60 | 0 |
| (15) | 78 | — | 1:5 | 90 | 2.6 |
| (16) | 105 | 61 | 1:3 | 90.47 | 6 |
| (17) | 45 | 65 | 1:2.7 | 82 | 0 |
| (18) | 43 | 63.2 | 1:2 | 90.6 | 2.6 |
| (19) | 59 | — | 1:3 | 84.2 | 1.6 |
| (3) | 390 | 65.5 | 1:8 | 83.8 | 3.2 |
| (20) | 57 | 69 | 1:2.6 | 54 | 1:5 |
| (21) | 72 | 70 | 1:2 | 80 | — |
| (22) | 28 | 67.1 | 1:4.6 | 89.3 | 7.1 |
| (23) | 117 | 67 | 1:3 | 69 | 1.7 |
| )24) | 22 | 64 | 1:3.5 | 77 | 4.5 |
| (25) | 181 | 70.5 | 1:4.5 | 88.4 | 1.6 |
| (26) | 82 | 63.8 | 1:3 | 74.6 | 5 |
| (27) | 100 | 49 | 1:2 | 45 | 2 |
| (28) | 68 | 69 | 1:4 | 70 | — |
| (7) | 66 | — | — | 80.3 | 1 |
| (29) | 108 | — | 1:4 | 83 | 6.4 |
| (30) | 45 | 62 | 1:2 | 82 | — |
| (31) | 75 | 62 | 1:5.25 | 98 | 1.6 |
| (32) | 328 | 45.01 | 1:2.6 | 100 | 1.8 |
| (33) | 48 | 71.7 | 1:1.8 | 66.6 | 12.5 |
| (34) | 67 | 63 | 1:2.4 | 57 | 5.9 |
| (35) | 100 | 68 | 1:1.4 | 26 | 10 |
| (36) | 110 | 72 | 1:3.2 | 70 | 2 |
| (37) | 100 | 65 | 1:3.34 | 78 | — |
| (38) | 69 | 68.2 | 1:1.76 | 60.4 | — |
| (39) | 20 | 74 | 1:2.3 | 70 | 5 |
| (40) | 112 | 65 | 1:3.5 | 92 | 4 |
| (41) | 287 | 70 | 1:3.7 | 78.4 | — |
| (42) | 315 | 50 | 1:2.5 | — | 1 |
| (43) | 131 | — | — | — | — |
| (44) | 134 | 67 | — | — | — |
| (9) | 49 | 63 | 1:3.45 | 93.5 | 12 |
| (45) | 53 | 65 | 1:4 | 66 | 4 |
| (8) | 68 | 72 | 1:2 | 76 | 0 |
| (46) | 94 | 80.5 | 1:2.9 | 60.6 | — |
| (47) | 21 | 75.9 | 1:4 | 85.7 | 4.76 |
| Total | 4375 | | | | |

that longstanding pancreaticocholedochal reflux in the presence of anomalous ductal union causes inflammation in the biliary duct and induces epithelial metaplasia[56].

*Benign gallbladder lesions* These lesions are generally classified into epithelial tumours (adenoma), mesenchymal tumours (fibroma, lipoma, haemangioma) and pseudotumours (cholesterol polyps, inflammatory polyps, adenomyoma); adenomas are classified as papillary, tubular or mixed[57]. According to Kozuka *et al.* adenomatous components are present in all the *in situ* carcinomas and 19 percent of invasive carcinomas and he suggests that carcinomas may arise directly from these adenomata[58]. There have been further developments in this area with the recent demonstration of two types of gallbladder adenocarcinoma, one being derived from ordinary gallbladder epithelium and the other from metaplastic epithelium. Either type may result from adenomata[59].

Based on these findings gallbladder carcinomas have been divided into metaplastic and non-metaplastic types according to the presence or absence of metaplastic markers, such as endocrine cells and lysozyme immunoreactivity in the tumour tissue. The metaplastic type is more commonly found in females and the survival rates are better. The modes of tumour spread also differ, the metaplastic type frequently showing lymphatic spread, whereas the non-metaplastic type usually spread by direct invasion. This classification correlates well with biological behaviour and might reflect the histogenesis of gallbladder carcinoma[60].

Adenomyomatosis of the gallbladder which is characterized by extensions of the mucosa into and through a thickened muscular wall also has malignant potential and 7 cases of gallbladder carcinoma arising in adenomyomatosis have been described[61]. In contrast there is little evidence that inflammatory or cholesterol polyps are even associated with malignant transformation[49].

*Diffuse calcification of the gallbladder* Diffuse calcification of the gallbladder is found in less than 0.1 percent of cholecystectomies and 95 percent of cases are associated with gallstones[5]. "Porcelain gallbladder" so called because of the characteristic blue discolouration and the brittle consistency of the gallbladder wall, is five times more frequent in women and is associated with gallbladder carcinoma in 10 to 25 percent of cases[62].

*Typhoid carriers* Chronic typhoid carriers die of hepatobiliary cancer six times more often than the matched controls. It has been suggested that a combination of bile stasis (secondary to bile duct obstruction) and alteration of bile salts by salmonella surviving in the gallbladder may be responsible[63].

*Xanthogranulomatous cholecystitis* This granulomatous inflammation caused by penetration of the gallbladder wall by bile lipids, is found in 0.7 to 1.8% of routine cholecystectomy specimens[64]. Xanthogranulomatous cholecystitis is also present in 8.6% of gallbladder carcinomas and is of importance because of the difficulty in differentiating the two conditions[65]. The occasional co-existence of these two conditions can further complicate the diagnostic problem.

*Occupation* Specific substances in the working environment (e.g. 3,3 dichorobenzidine) directly or in combination with other chemicals have been shown to be partly responsible for the development of carcinoma of the gallbladder, bile ducts and liver. This is a particular problem amongst rubber workers[5,66].

*Chronic inflammatory bowel disease* Inflammatory bowel disease is associated with an increased risk of carcinoma of the biliary tree and in 13 percent of these cases, the tumour originates in the gallbladder. The risk is highest in those with a long history of colitis, especially if there is total involvement of the colon[67]. In these patients carcinoma occurs 10 to 15 years before the peak incidence in the general population[49].

*Previous biliary surgery* In several uncontrolled studies patients who have undergone previous operations, particularly cholecystostomy, appear to have a higher incidence of gallbladder carcinoma. It is suggested that these tumours can occur anywhere from 1.3 to 50 years after the original procedure[49,68].

### CLINICAL FEATURES

Carcinoma of the gallbladder, despite being the most common malignancy of the biliary tract and the fifth most common gastrointestinal malignancy, because of its nonspecific clinical signs and symptoms, is nearly always diagnosed at a late stage[69]. The clinical picture initially may mimic benign gallbladder disease until there is invasion or spread to adjacent organs. Even when spread has occurred, the mean correct pre-operative rate of diagnosis is only about 10% (4.3 to 19%)[3,11,15,23,38,45,49].

In the review by Alborez-Saavedra and Henson the most common initial complaint was pain in the right upper quadrant (75% of patients) which was continuous or intermittent[5]. Jaundice due to obstruction of the common bile duct or to liver metastases occurred in 30 to 60 percent of cases and was usually an ominous sign, indicating nonresectability of lesions. Less common signs were ascites (20%), duodenal obstruction (10%), palpable mass, hepatomegaly, anorexia, nausea, vomiting and weight loss[5]. These findings are similar to those reported in the collective reviews of Piehler and of Vaittinen and Gupta's review of 328 cases of primary carcinoma of the gallbladder from India[3,32,49] (See Table 2). In Vaittinen's review of 3958 patients in 89 series, pain was present in 79% of patients the duration of symptoms often being difficult to establish due to previous or coexisting symptoms of biliary disease[4]. Whilst the duration of symptoms in this series varies from a matter of hours to more than 40 years[9,20,27,31,32,37], the usual time before presentation is between 3.5 and 5.5 months[11]. Signs and symptoms present at the resectable stage of gallbladder carcinoma are so similar to those of non-malignant diseases of the organ that late diagnosis and a dismal 5 year survival rate often results.

### DIAGNOSTIC METHODS

Haematological and biochemical parameters are of almost no value in the investigation of malignant gallbladder disease and it is essential if there is the slightest suspicion to study gallbladder morphology. The investigations usually employed in this respect are cholecystography, ultrasound examination (US), computed tomography (CT) and angiography[34].

Cholecystography is unreliable because filling of the gallbladder is very variable with or without gallstones[70]. In contrast, US examination and computed tomography seem to increase the likelihood of accurate pre-operative diagnosis and three primary tumour patterns have recently been described; a **mass replacing** the gallbladder in 40 to 65% of cases, **local or diffuse thickening** of the gallbladder wall in 20 to 30% of cases and the least common form of gallbladder carcinoma (15 to 25% of cases) is an **intraluminal mass**[71,72,73]. In the latter group carcinomas tend to expand into the lumen of the gallbladder before invading the wall resulting in an improved prognosis but of course also increased diagnostic difficulties. All these investigations, however, frequently fail to differentiate gallbladder wall thickening due to inflammatory disease from that due to tumour (although in the latter the wall is typically thicker and more irregular) and it is important in cases of polypoid tumours over 5 mms in diameter or abnormal thickening of the gallbladder wall to have a high index of suspicion[71,73]. Non-visualisation of the gallbladder by US or computed tomography in patients with a mass in the right upper quadrant is also said to be suggestive of gallbladder carcinoma[73]. In difficult cases the use of higher frequency transducers, standoff pads (with redirection of scanning angle if the anterior wall is obscured by reverberations) and decubitus, erect and prone views (to distinguish sludge from a mass) are all said to improve the diagnosis of early lesions[74].

In a review of 67 patients by Tashiro, a correct diagnosis was made in 58.3% of the patients examined by angiography and in his series it was the most useful diagnostic measure to detect the disease at a resectable stage[34]. Coeliac or selective hepatic angiography can be used to demonstrate malignancies of the gallbladder when the disease is still localised to the gallbladder wall. In these cases enlarged cystic or hepatic arteries, infiltration and cutoff of the cystic artery or its branches, neovasculature of the cystic wall and irregular thickness of the gallblader wall in the venous phase, are all important criteria of malignancy[70]. Very occasionally endoscopic retrograde cholangiopancreatography (ERCP) can also be useful showing an irregular filling defect within the gallbladder or obstruction of the common hepatic duct close to the hilus with a failure of the gallbladder to fill. Generally, however, despite all these advances, correct, definite diagnoses are rarely made[3].

### TREATMENT OPTIONS

No treatment option has significantly altered the relentless and fatal course in the majority of cases of carcinoma of the gallbladder, although a few long term survivors have been reported. This is due not to the natural history of this cancer, but to diagnostic problems (vide supra) that allow involvement of the liver and porta hepatis by the time of operation. As a consequence it is often only possible to perform biopsies and palliative bypass procedures. The various treatments (surgical and non surgical) are shown in Table 3.

**Table 3** Available treatment options

| *Modalities* | | *References* |
|---|---|---|
| *Surgical* | | |
| a) Curative: | – Cholecystectomy | (7, 77, 80) |
| | – Extended cholecystectomy | (78, 79, 80) |
| | – Radical surgery | (6, 81) |
| b) Palliative: | – Biopsy | (2) |
| | – Biliary-enteric bypass | (2) |
| | – Surgery + Radiotherapy | (85, 86, 87) |
| | – Surgery + Radiotherapy + Chemotherapy | (85) |
| *Non-Surgical* | | |
| a) Radiotherapy | | (82, 83) |
| b) Chemotherapy | | (84) |

Before attempting critical analysis of any of the available treatments, it is important to consider known prognostic factors. Of these **the depth of invasion of the gallbladder wall and histologic grade** correlate well with survival[7,75] and in a recent review of 21 patients who lived 5 or more years, tumours were well differentiated carcinoma in 10, moderately differentiated in four and mixed in six[76]. It has also been shown that the **papillary form** of gallbladder carcinoma is associated with a better patient survival than the nodular or infiltrative form. This is probably because the papillary form is predominantly limited to the mucosa and has a lower cellular malignancy than those tumours which extend to the muscularis, subserosa and serosa (as evaluated by examination of nuclear DNA patterns in cancer cells from these tumours)[75].

*Surgical Treatments* It is generally accepted that simple classifications based on staging, grading or a combination of these two factors can be applied to surgically removed gallbladder carcinomas and produces useful information regarding prognosis. In more than 75% of cases, simple cholecystectomy is performed for presumed benign disease but histological examination of the specimen shows carcinoma. In a study of 32 patients with only mucosal or submucosal involvement had a 63.6% five year survival and 45.5% 10 year survival[77]. In Nevin's similar series, 86% of patients having only intramucosal involvement lived more than five years[7], and it seems that patients with these Stage I and II carcinomas of the gallbladder are usually cured by simple cholecystectomy. In contrast Stages III and IV have a poor outlook although the prognosis may be improved by an extended or radical cholecystectomy, with or without right hepatic lobectomy and adequate nodal dissection. Stage V is so advanced at the time of diagnosis that surgical treatment does not influence the outcome[7]. The various modes of spread of gallbladder carcinoma have led some authors to believe that even with early stages of the disease, simple cholecystectomy is an inadequate surgical practice. Consequently it is often suggested that even in the incidentally found carcinoma, an "adequate" operation must include cholecystectomy, wedge resection of the liver with a margin of free tissue and adequate nodal dissection. For more advanced disease with liver involvement some authors suggest that as well as cholecystectomy a right or extended hepatic lobectomy, adequate nodal dissection including the coeliac nodes and resection of neighbouring involved organs (whenever possible) should be undertaken[78,79]. There is, however, no clear concensus on this point and even recently it has been suggested that carcinomas of the gallbladder limited to the mucosa can be treated only by cholecystectomy but for tumours involving the subserosa or muscular layers, re-operation is advised (liver wedge resection and nodal dissection) because of the increased incidence of lymphatic and liver involvement amongst these patients[80].

When carcinoma of the gallbladder spreads to the liver in a high percentage of cases it does so in a localised rather than disseminated form, involves the pericholedochal nodes (in the lesser omentum and behind the first part of the duodenum) in 20% of cases, the various neighbouring organs and abdominal wall in about 20% of cases and less commonly the extraheptic biliary ducts. A truly curative supraradical approach would therefore have to include cholecystectomy, adequate resection of the liver, meticulous dissection of the known areas of lymphatic drainage and resection of the involved ducts and neighbouring organs or abdominal wall[6]. Aggressive surgery for carcinoma of the gallbladder including an extended right lobectomy or pancreaduodenectomy or extended lobectomy of the liver combined with pancreatoduodenectomy or an extended right lobectomy combined with portal resection has also been recently undertaken but further follow up is obviously needed to assess the long term value of these procedures[81]. Whether radical treatment is undertaken or not, however, patients with carcinomas of the gallbladder can usually be offered palliative treatment and even when they are jaundiced, biliary enteric bypass using the left hepatic ductal system (Segment 3 approached via the umbilical fissure) can be employed[3].

*Non surgical treatment* Primary carcinoma of the gallbladder remains resectable in only 25 percent of cases[1]. Consequently, non-interventional treatments have been investigated particularly external irradiation alone or in combination with chemotherapy. A 20% response of gallbladder carcinoma to radiation therapy has been reported in one series (900 rads per week with a total dose of 4,500 rads)[82]. Other groups have not been so encouraged and in one series where 4,500 to 5,000 rads (in 180–200 rad fractions) was given, to the tumour and regional lymph nodes, the outcome was very poor with deaths at 5.$\frac{1}{2}$, 6, 9 and 10 months from the date of diagnosis[83]. While further evaluation of this mode of treatment is obviously needed it seems very unlikely that used alone it will be of any but palliative value. The situation is similar for combination chemotherapy. With fluorouracil, doxorubicin and methotrexate, in one series there was a partial response in 4 out of 13 patients (31%) with a median duration of 8.5 months[84]. In another similar study a 29% partial response was also obtained with a four-drug combination of streptozocin, semustine, fluorouracil and vincristine with 18% minor remission and an 11 month median survival rate[84]. Thus while chemotherapy may produce temporary partial remissions, there is no evidence to date of any long term survivors. There is also recent limited experience regarding adjuvant postoperative external irradiation with or without chemotherapy. It seems that radiotherapy may increase survival where no curative surgery can be performed[85,86,87], but there is no clear difference in survival between patients and controls with regard to any regimens of chemotherapy in this period[85].

### CONCLUSION

Primary carcinoma of the gallbladder is a rare malignancy with a dismal prognosis, mainly due to its non-specific clinical symptoms and signs, and the lack of a reliable diagnostic test. The result is almost invariably a delay before effective treatment, which allows vital adjacent organs to become involved. Although ultrasound scans and computerised tomography have a high resolution in biliary tract pathology, they have not yet proved satisfactory in the pre-operative diagnosis of gallbladder carcinoma. Selective hepatic angiography on the other hand has given encouraging results in the differential diagnosis of gallbladder abnormalities but further evaluation of this invasive technique is required. While initial experience suggests that digital subtraction angiography may become a valuable tool in the pre-operative assessment, to date, a high index of clinical suspicion remains the mainstay of early diagnosis and a consequently better prognosis, although naturally a small number of cases will be discovered incidentaly during ultrasound examinations.

In respect of "curative surgical management" evidence so far is generally in favour of accepting simple cholecystectomy when the tumour is confined to the mucosa but re-operating to do an extended or radical cholecystectomy when the tumour extends through the gallbladder wall but has not yet invaded the serosa; in the latter situation, aggressive surgery (although advocated by a few enthusiasts) does not confer reproducible or consistent improvements in quality of life or survival.

The association of gallstones and primary carcinoma of the gallbladder has often raised the question of prophylactic cholecystectomy. Mortality from gallbladder carcinoma has declined markedly in England, Wales, Scotland, the United States and Canada, but has risen by $\frac{1}{3}$ in Sweden over the last 20 years. These findings correlate with cholecystectomy rates[88], and furthermore every 100 cholecystectomies performed reduces the number of deaths from carcinoma of the gallbladder by one. It is always worth remembering, however, that routine cholecystectomy in patients with asymptomatic cholelithiasis which carries an associated morbidity and mortality should be balanced against the risk of surgery and the general medical condition of the patients.

These observations clearly leave one important question unanswered. What will be the effect of gallstone treatments that leave an intact (and damaged?) gallbladder mucosa *in situ* for years after the treatment is finished? On this point, one can only speculate but careful follow up of patients after extracorporeal shock wave (lithotripsy) or percutaneous gallstone extraction is going to be essential (although the development of carcinoma has been shown to be related to gallstone diameter). Furthermore, these fears obviously make treatments which include ablation of the mucosa increasingly attractive in terms of long term safety.

### *References*

1 Adson, M.A. (1973) Carcinoma of the gallbladder. *Surg. Clin. North Am.*, **53**, 1203–1216
2. Muir, I.M. and Morris, D.L. (1986) Carcinoma of the gallbladder. *Br. J. Hosp. Med.*, **36**, 278–280
3. Blumgart, L.H. and Imrie, C.W. (1985) Carcinoma of the gallbladder. In: Wright, R, G.H. Millward-Sadler, K.G.M.M. Alberti and S. Karran (eds): *Liver and Biliary Disease*. London, WB Saunders, pp. 1495–1498
4. Vaittinen, E. (1970) Carcinoma of the gallbladder; a study of 390 cases diagnosed in Finland 1953–1967. *Ann. Chir. Gynaecol. Fenn.* **59**, (Suppl.168) :7-81
5. Albores-Saavedra, J. and Henson, D.E. (1986) Tumors of the gallbladder and extrahepatic bile ducts. In: *Atlas of tumor pathology. Fasc 22.* Washington DC. Armed Forces Institute of Pathology. pp. 17–123
6. Fahim, R.B., McDonald, J.R., Richards, J.C. and Ferris, D.O. (1962) Carcinoma of the gallbladder; a study of its modes of spread. *Ann. Surg.*, **156**, 114–124
7. Nevin, J.E., Moran, T.J., Kay, S. and King, R. (1976) Carcinoma of the gallbladder; staging, treatment and prognosis. *Cancer*, **37**, 141–148
8. Tarpila, E., Borch, K., Kullman, E. and Liedberg, G. (1988) Gallbladder cancer; current status in clinical practice. *Eur. J. Surg. Oncol.* **14**, 51–54
9. Roberts, J.W. and Daugherty, S.F. (1986) Primary carcinoma of the gallbladder. *Surg. Clin. North Am.* **66**, 743–749
10. Tabet, B.J. (1960) Primary carcinoma of the gallbladder; report of 47 cases. *Am. J. Surg.*, **100**, 365–371
11. Strauch, G.O. (1960) Primary carcinoma of the gallbladder. *Surgery*, **47**, 368–383
12. Gerst, P.H. (1961) Primary carcinoma of the gallbladder; a thirty year summary. *Ann. Surg.*, **153**, 369–372
13. Bossart, P.A., Patterson, A.H. and Zintel, H.A. (1962) Carcinoma of the galbladder; a report of 76 cases. *Am. J. Surg.*, **103**, 366–369
14. Robertson, W.A. and Carlisle, B.B. (1967) Primary carcinoma of the gallbladder; review of 52 cases. *Am. J. Surg.*, **113**, 738–742
15. Litwin, M.S. (1967) Primary carcinoma of the gallbladder; a review of 78 patients. *Arch. Surg.*, **95**, 236–240

16. Warren, K.W., Hardy, K.J. and O'Rourke, M.G.E. (1968) Primary neoplasia of the gallbladder. *Surg. Gynecol. Obstet.*, **126**, 1036–1040
17. Andrews, E.C.; Bennett, D.E. and Arhelger, R.B. (1969) Carcinoma of the gallbladder; report of 45 cases. *South. Med. J.*, **62**, 573–578
18. Tanga, M.R. and Ewing, J.B. (1970) Primary malignant tumors of the gallbladder; report of 43 cases. *Surgery*, **67**, 418–426
19. Hardy, M.A., and Volk, H. (1970) Primary carcinoma of the gallbladder; a ten year review. *Am. J. Surg.*, **120**, 800–803
20. Solan, M.J. and Jackson, B.T. (1971) Carcinoma of the gallbladder; a clinical appraisal and review of 57 cases. *Br. J. Surg.*, **58**, 593–597
21. Holmes, S.L. and Mark, J.B.D. (1971) Carcinoma of the gallbladder. *Surg. Gynecol. Obstet.*, **133**, 561–564
22. Klein, J.B. and Finck, F.M. (1972) Primary carcinoma of the gallbladder; review of 28 cases. *Arch. Surg.*, **104**, 769–772
23. Beltz, W.R. and Condon, R.E. (1974) Primary carcinoma of the gallbladder. *Ann. Surg.*, **180**, 180–184
24. Balaroutsos, C., Bastounis, E., Karamanakos, P. and Golematis, B. (1974) Primary carcinoma of the gallbladder; analysis of 22 cases. *Am. Surg.*, **40**, 605–608
25. Ohlsson, E.G. and Aronsen, K.F. (1974) Carcinoma of the gallbladder; a study of 181 cases. *Acta Chir. Scand.*, **140**, 475–480
26. Moossa, A.R., Anagnost, M., Hall, A.W., Moraldi, A. and Skinner, D.B. (1975) The continuing challenge of gallbladder cancer; survey of thirty years' experience at the University of Chicago. *Am. J. Surg.*, **130**, 57–62
27. Prakash, A.T.M., Sharma, L.K. and Pandit, P.N. (1975) Primary carcinoma of the gallbladder. *Br. J. Surg.*, **62**, 33–36
28. Donaldson, L.A. and Busuttil, A. (1975) A clinicopathological review of 68 carcinomas of the gallbladder. *Br. J. Surg.*, **62**, 26–32
29. Richard, P.F. and Cantin, J. (1976) Primary carcinoma of the gallbladder; study of 108 cases. *Can. J. Surg.*, **19**, 27–32
30. Weiskopf, J. and Esselstyn, C.B. (1976) Carcinoma of the gallbladder; a 25 year review. *Am. J. Gastroenterol.*, **65**, 522--527
31. DoCarmo, M., Perpetuo, M.O., Valdivieso, M., Heilbrun, L.K., Nelson, R.S., Connor, T. and Bodey, G.P. (1978) Natural history study of gallbladder cancer. *Cancer*, **42**, 330–335
32. Gupta, S., Udupa, K.N. and Gupta, S. (1980) Primary carcinoma of the gallbladder; a review of 328 cases. *J. Surg. Oncol.*, **14**, 35–44
33. Shieh, C.J., Dunn, E. and Standard, J.E. (1981) Primary carcinoma of the gallbladder; a review of a 16 year experience at the Waterbury Hospital Health Center. *Cancer* **47**, 996–1004
34. Tashiro, S., Konno, T., Mochinaga, M., Watanable, E., Murate, E., Vemura, K. and Yokoyama, I. (1981) Primary carcinoma of the gallbladder; a review of 67 cases. *Kumamoto Med. J.*, **34**, 1–12
35. Koo, J., Wong, J., Cheng, F.C.Y. and Ong, G.B. (1981) Carcinoma of the gallbladder. *Br. J. Surg.*, **68**, 161–165
36. Kelly, T.R. and Chamberlain, T.R. (1982) Carcinoma of the gallbladder. *Am. J. Surg.*, **143**, 737–741
37. Wanebo, H.J., Castle, W.N. and Fechner, R.E. (1982) Is carcinoma of the gallbladder a curable lesion? *Ann. Surg.*, **195**, 624–630
38. Hamrick, R.E., Liner, F.J., Hastings, P.R. and Cohn, I. (1982) Primary carcinoma of the gallbladder. *Ann. Surg.*,, **195**, 270–273
39. Klamer, T.K. and Max, M.H. (1983) Carcinoma of the gallbladder. *Surg. Gynecol. Obstet.*, **156**, 641–645
40. Morrow, C.E., Sutherland, D.E.R., Florack, G., Eisenberg, M.M. and Grage, T.B. (1983) Primary gallbladder carcinoma; significance of subserosal lesions and results of aggressive surgical treatment and adjuvant chemotherapy. *Surgery* **94**, 709–713
41. Sons, H.U., Borchard, F. and Joel, B.S. (1985) Carcinoma of the gallbladder; autopsy findings in 287 cases and review of the literature. *J. Surg. Oncol.*, **28**, 199-206
42. Shukla, V.K., Khandelwal, C., Roy, S.K. and Vaidya, M.P. (1985) Primary carcinoma of the gallbladder; a review of a 16 year period at the University Hospital. *J. Surg. Oncol.*, **28**, 32–35
43. Lowenfels, A.B., Lindstrom, C.G., Conway, M.J. and Hastings, P.R. (1985) Gallstones and risk of gallbladder cancer. *J.N.C.I.* **75**, 77–80
44. Whetstone, M.R., Saltzstein, E.C. and Mercer, L.C. (1986) Demographic characteristics of gallbladder cancer in an area endemic for biliary calculi. *Am. J. Surg.*, **152**, 728–730

45. White, K., Kraybill, W.G. and Lopez, M.J. (1988) Primary carcinoma of the gallbladder; TNM staging and prognosis. *J. Surg. Oncol.*, **39**, 251–255
46. Kimura, W., Shimada, H., Kuroda, A. and Morioka, Y. (1989) Carcinoma of the gallbladder and extrahepatic bile duct in autopsy cases of the aged with special reference to its relationship to gallstones. *Am. J. Gastroenterol.*, **84**, 386–390
47. Paraskevopoulos, J.A., Dennison, A.R. and Johnson, A.G. (1991) Primary carcinoma of the gallbladder; a ten year experience. In Press.
48. Gibney, E.J. (1990) Asymptomatic gallstones. *Br. J. Surg.*, **77**, 368–372
49. Piehler, J.M. and Crichlow, R.W. (1978) Primary carcinoma of the gallbladder. *Surg. Gynecol. Obstet.*, **147**, 929–942
50. Maringhini, A., Moreau, J.A., Melton, L.J., Hench, V.S., Zinsmeister, A.R. and Dimagno, E.P. (1987) Gallstones, gallbladder cancer and other gastrointestinal malignancies. *Ann. Int. Med.*, **107**, 30–35
51. Diehl, A.K. (1982) Silent gallstones; the doctor's dilemma. *Compr. Ther.*, **8**, 62–68
52. Diehl, A.K. (1983) Gallstone size and the risk of gallbladder cancer. *JAMA*, **250**, 2323–2326
53. Lowenfels, A.B., Walker, A.M., Althaus, D.P., Townsend, G. and Domellof, L. (1989) Gallstone growth, size and risk of gallbladder cancer; an interracial study. *Inter. J. Epidemiol.*, **18**, 50–54
54. Flanigan, D.P. (1975) Biliary cysts. *Ann. Surg.*, **182**, 635–643
55. Tsuchiya, R., Harada, N., Ito, T., Furukawa, M., Yoshihiro, I., Kusano, T. and Uchimura, M. (1977) Malignant tumours in choledochal cysts. *Ann. Surg.*, **186**, 22–28
56. Miyazaki, K., Date, K., Imamura, S., Ogawa, Y. and Nakayama, F. (1989) Familial occurrence of anomalous pancreaticobiliary duct union associated with gallbladder neoplasms. *Am. J. Gastroenterol.*, **84**, 176–181
57. Aldridge, M.C. and Bismuth, H. (1990) Gallbadder cancer; the polyp cancer sequence. *Br. J. Surg.*, **77**, 363–364
58. Kozuka, S., Tsubone, M., Yasui, A. and Hachisuka, K. (1982) Relation of adenoma to carcinoma in the gallbladder. *Cancer* **50**, 2226–2234
59. Yamamoto, M., Nakajo, S. and Tahara, E. (1989) Histogenesis of well-differentiated adenocarcinoma of the gallbladder. *Path. Res. Pract.*, **184**, 279–286
60. Yamamoto, M., Nakajo, S. and Tahara, E. (1989) Carcinoma of the gallbladder; the correlation between histogenesis and prognosis. *Virchows Archiv. A Pathol. Anat.*, **414**, 83–90
61. Paraf, F. and Potet, F. (1988) Gallbladder carcinoma arising in adenomyomatosis (Letter). *Am. J. Gastroenterol.*, **83**, 1439
62. Berk, R.N., Armbuster, T.G. and Saltzstein, S.L. (1973) Carcinoma in the porcelain gallbladder. *Radiology*, **106**, 29–31
63. Welton, J.C., Marr, J.S. and Friedman, S.M. (1979) Association between hepatobiliary cancer and typhoid carrier state. *Lancet*, **1**, 791–794
64. Roberts, K.M. and Parsons, M.A. (1988) Simultaneous xanthogranulomatous cholecystitis and primary adenocarcinoma of the gallbladder (Letter). *Histopathology*, **13**, 708
65. Benbow, E.W. (1989) Xanthogranulomatous cholecystitis associated with carcinoma of the gallbladder. *Postgrad. Med. J.*, **65**, 528--531
66. Mancuso, T., Brennan, M.J. (1970) Epidemiological considerations of cancer of the gallbladder, bile ducts and salivary glands in the rubber industry. *J. Occup. Med.*, **12**, 333–341
67. Joffe, N. and Antonioli, D.A. (1981) Primary carcinoma of the gallbladder associated with chronic inflammatory bowel disease. *Clin. Radiology*, **32**, 319–324
68. Malone, D.E. and Burhenne, H.J. (1989) Advantages and disadvantages of the newer interventional procedures for the treatment of cholecystolithiasis. *Hepato-gastroenterol.*, **36**, 317–326
69. Spinale, R.C. and Meeker, J.F. (1989) Carcinoma of the gallbladder. *JAOA*, **89**, 625–629
70. Pettersson, H. (1974) Carcinoma of the gallbladder; a review of 158 cases. *Acta Radiol. Diag.*, **15**, 225–236
71. Lane, J., Buck, J.L. and Zeman, R.K. (1989) Primary carcinoma of the gallbladder; a pictorial essay. *Radiographics*, **9**, 209–228
72. Koga, A., Yamauchi, S., Izumi, Y. and Hamanaka, N. (1985) Ultrasonographic detection of early and curable carcinoma of the gallbladder. *Br. J. Surg.*, **72**, 728–730
73. Weiner, S.N., Koenigsberg, M., Morehouse, H. and Hoffman, J. (1984) Sonography and computed tomography in the diagnosis of carcinoma of the gallbladder. *AJR*, **142**, 735–739
74. O'Keefe, F., Lorigan, G. and Butler, F. (1989) Ultrasound findings in carcinoma of the gallbladder. *Ir. J. Med. Sci.*, **158**, 48–49

75. Ouchi, K., Owada, Y., Matsuno, S. and Sato, T. (1987) Prognostic factors in the surgical treatment of gallbladder carcinoma. *Surgery,* **101**, 731–737
76. Appleman, R.M., Morlock, C.G., Dahlin, D.C. and Adson, M.A. (1963) Long term survival in carcinoma of the gallbladder. *Surg. Gynecol. Obstet.,* **117**, 459–464
77. Bergdahl, L. (1980) Gallbladder carcinoma first diagnosed at microscopic examination of gallbladders removed for presumed benign disease. *Ann. Surg.,* **191**, 19–22
78. Fahim, R.B., Ferris, D.O. and McDonald, J.R. (1963) Carcinoma of the gallbladder; an appraisal of its surgical treatment. *Arch. Surg.,* **86**, 176–183
79. Isman, H. and Bourgeon, R. (1986) A curative surgical approach to gallbladder carcinoma in its early stages. *Ital. J. Surg. Sci.,* **16**, 117–122
80. DeAretxabala, X., Roa, I., Araya, J.C., Burgos, L., Flores, P., Huenchullan, I. and Miyazaki, I. (1990) Operative findings in patients with early forms of gallbladder cancer. *Br. J. Surg.,* **77**, 291–293
81. Nakamura, S., Sakaguchi, S., Suzuki, S. and Muro, H. (1989) Aggressive surgery for carcinoma of the gallbladder. *Surgery,* **106**, 467–473
82. Smoron, G.L. (1977) Radiation therapy of carcinoma of gallbladder and biliary tract. *Cancer,* **40**, 1422–1424
83. Buskirk, S.J., Gunderson, L.L., Adsom, M.A., Martinez, A., May, G.R., McIlath, D.C., Nagorney, D.M., Edumondson, G.K., Bender, C.E. and Martin, J.K. (1984) Analysis of failure following curative irradiation of gallbladder and extraheptic bile duct carcinoma. *Int. J. Radiat. Oncol. Biol. Phys.* **10**, 2013–2023
84. Wright, J.C. (1986) Update in cancer chemotherapy; gastro-intestinal cancer, cancer of the small intestines, gallbladder, liver and oesophagus. *J. Nat. Med. Assoc.,* **78**, 753–766
85. Houry, S., Schlienger, M., Huguier, M., Lacaine, F., Penne, F. and Laugier, A. (1989) Gallbadder carcinoma; role of radiation therapy. *Br. J. Surg.,* **76**, 448–450
86. Bosset, J.F., Mantion, G., Gillet, M., Pelissier, E., Boulenger, M., Maingon, P., Corbion, O. and Schraub, S. (1989) Primary carcinoma of the gallbladder; adjuvant postoperative external irradiation. *Cancer,* **64**, 1843–1847
87. Treadwell, T.A. and Hardin, W.J. (1976) Primary carcinoma of the gallbladder; the role of adjunctive therapy in its treatment. *Am. J. Surg.,* **132**, 703–706
88. Diehl, A.K. and Beral, V. (1981) Cholecystectomy and changing mortality from gallbladder cancer. *Lancet,* **2**, 187–189

*(Accepted by S. Bengmark 27 March 1991)*

